# Research progress and application of artificial intelligence in thyroid associated ophthalmopathy

**DOI:** 10.3389/fcell.2023.1124775

**Published:** 2023-01-24

**Authors:** Jiale Diao, Xinxin Chen, Ya Shen, Jian Li, Yuqing Chen, Linfeng He, Sainan Chen, Pei Mou, Xiaoye Ma, Ruili Wei

**Affiliations:** Department of Ophthalmology, Changzheng Hospital of Naval Medicine University, Shanghai, China

**Keywords:** thyroid-associated ophthalmopathy, artificial intelligence, deep learning, automated diagnosis, facial images

## Abstract

Thyroid-associated ophthalmopathy (TAO) is a complicated orbitopathy related to dysthyroid, which severely destroys the facial appearance and life quality without medical interference. The diagnosis and management of thyroid-associated ophthalmopathy are extremely intricate, as the number of professional ophthalmologists is limited and inadequate compared with the number of patients. Nowadays, medical applications based on artificial intelligence (AI) algorithms have been developed, which have proved effective in screening many chronic eye diseases. The advanced characteristics of automated artificial intelligence devices, such as rapidity, portability, and multi-platform compatibility, have led to significant progress in the early diagnosis and elaborate evaluation of these diseases in clinic. This study aimed to provide an overview of recent artificial intelligence applications in clinical diagnosis, activity and severity grading, and prediction of therapeutic outcomes in thyroid-associated ophthalmopathy. It also discussed the current challenges and future prospects of the development of artificial intelligence applications in treating thyroid-associated ophthalmopathy.

## Introduction

Artificial intelligence (AI) has gradually become a part of each aspect of our lives, especially medicine, with the rapid development of computer technologies and smart devices. This term did not emerge recently but was first proposed at a conference in 1956 (Russell and Norvig, 2010). The early achievement of AI applications in medicine was the automated recognition of electrocardiograms, which was based on programmed medical knowledge (Kundu et al., 2000). Machine learning (ML), a subfield of computer science, endowed AI with the ability to independently discern patterns from data. The training set, containing several inputs and relevant outputs, is critical for ML methods to analyze the underlying patterns, which help obtain correct outputs from new inputs (Deo, 2015). Further, deep learning (DL) has given a major boost to the AI renaissance in recent decades. DL methods generally build an artificial neural network with many layers to analyze colossal datasets, such as numerous medical images (LeCun et al., 2015; Schmidhuber, 2015).

The application of integrated AI-ML-DL algorithms, combined with advanced medical imaging and data transmission systems, has grown rapidly in the medical field, such as ophthalmic healthcare (Balyen and Peto, 2019). For instance, diabetic retinopathy (DR) can be detected by screening the retina using fundus photography and optical coherence tomography as a representative chronic ocular disease. It was found that multiple AI applications in retinal images had significant benefits in the early detection of DR (Gulshan et al., 2016; Ting et al., 2017; Tufail et al., 2017). Recent studies also revealed that the detection of glaucoma could be promoted using AI-ML-DL algorithms with high accuracy, sensitivity, and specificity (Li et al., 2018a; Devalla et al., 2018).

Thyroid-associated ophthalmopathy (TAO), an intricate autoimmune disease, is associated with the highest incidence of the orbital disorder in adults, affecting approximately 2.9 men and 16 women per hundred thousand people every year (Bartley et al., 1995; Wiersinga and Bartalena, 2002). Severe cases tend to develop in male and older patients, accompanied by disfiguring proptosis and optic neuropathy (Bahn, 2010). The clinical manifestations of TAO include chemosis, eyelid retraction, exophthalmos, periorbital pain, and strabismus. Besides, the course of the disease is described as Rundle’s curve, which is composed of a one- to 3-year active phase and a subsequent chronic stable phase (Khong et al., 2016). This characteristic of TAO can be graded according to the clinical activity score and the severity grading identified by European Group on Graves’ orbitopathy (EUGOGO) (Bartalena et al., 2021). Variations of patterns in patients make TAO diagnosis, evaluation, and management challenging, which immensely depend on the profession and experience of well-trained ophthalmologists. AI applications may act as a supporting role in TAO clinical practice.

This review summarized the research progress and prospective application of AI in TAO diagnosis and management. The available studies focused on the identification of characteristic signs, disease grades, and dysthyroid optic neuropathy (DON); prediction of TAO progression; therapeutic response to glucocorticoids (GCs) and decompression surgery; and even protocol formulation of orbital radiotherapy. Given the prosperity of this “Big Data” era, we believe that this review could comprehend the current achievements and accelerate the promising AI applications in clinical practice, which may help ophthalmologists and endocrinologists with limited experience.

## Application of AI algorithms in detecting the signs and symptoms of TAO

As mentioned earlier, TAO generally starts with an active course. In this stage, patients suffer from ocular pain, redness and swelling of the conjunctiva and eyelids, and, most importantly, progressive proptosis and vision loss (Mourits et al., 1989). Early intervention, such as GC pulse therapy, can lead to premature termination of the active course and the start of a stable phase (Kauppinen-Mäkelin et al., 2002). Therefore, the early and accurate diagnosis of TAO can benefit the following management and prognosis. However, a large proportion of patients with TAO do not approach the department of ophthalmology, but the department of endocrinology, at the first visit because of thyroid dysfunction. Also, a few symptoms and signs of TAO are insidious enough to be missed during the examination. Thus, an automated diagnostic system assisted by AI algorithms can significantly increase the clinical efficiency of TAO diagnosis.

Grus et al. (1998) first tested an artificial neural network (ANN) in TAO. This ANN, a kind of probalistic neural network, contained input, pattern, summation and output layers, which could recognize the possible class of samples after training, thus possessing the diagnostic value. The sera samples were collected from patients with or without TAO (*n* = 16:11), Western blot analysis was performed, and densitometric data were collected. After training, 96.3% of test samples were correctly classified using an ANN, exceeding the multivariate statistical technique with 85% accuracy. This initial research enlightened the diagnostic potential provided by AI methods in TAO, though the autoantibodies detected in this study were not useful in TAO diagnosis. A few years later, Salvi et al. focused on the clinical signs and specialist examination of patients with TAO in two analogical studies (Salvi et al., 2002a; Salvi et al., 2002b). The samples were both divided into two groups based on disease progression. The ANN applicated in two studies was a back-propagation model used for the classification and progression prediction of TAO, which was constructed with 13 input variables derived from ophthalmic examinations. The accuracy of classification and progression prediction was 78.3%–86.2% and 67%–69.2%, respectively. As to the fundamentals of AI application in TAO diagnosis, these DL methods still need manual parameters measured by ophthalmologists or physicians.

After 2 decades of technological updating, advanced face recognition and automated image processing systems have increased the possibility for AI application in TAO. An intelligent diagnostic system for TAO was invented using multiple task-specific models based on facial images (Huang et al., 2022). Briefly, an entire facial image was analyzed and cropped into the eye part using Module I. Ocular dyskinesia and special signs of TAO were subsequently detected using Modules II and III. This study recruited 21,840 images from 1560 patients, of which 20% were used as the test set. The accuracy of eye location and cornea and sclera segmentation, conducted using Modules I and II, was 0.98, 0.93, and 0.87, respectively. The area under the receiver-operating characteristic curve (AUROC), sensitivity, and specificity of detecting signs were 0.93, 87%, and 88% for eyelid retraction; 0.90, 79%, and 86% for eyelid edema; 0.94, 89%, and 90% for eyelid congestion; 0.91, 83%, and 85% for conjunctival congestion; and 0.91, 85%, and 79% for ocular dyskinesia, respectively. Besides, the AUROC of DL networks (ResNet-50, ResNet-101, and InceptionV3) was 0.91, 0.92, and 0.89, respectively. Compared with previous models, this automated diagnostic system detected TAO signs highly accurately just with facial images. Besides, this system could also be loaded into mobile devices, thus showing the potential to help patients in areas lacking veteran ophthalmologists and medical resources.

Karlin et al. (2022) developed another AI platform based on a DL model to identify TAO using ocular photographs. The training set contained 1944 facial images, and the testing depended on additional 344 photographs. In line with the testing results, the accuracy, specificity, precision, recall, and F1 score of the proposed platform reached 89.2%, 86.9%, 79.7%, 93.4%, and 86.0%, respectively. The specific signs of TAO were not separated but integrated into a component model, thus generating heatmaps to present the pathological regions in facial images. This DL model was also compared with a cohort of ophthalmologists in the diagnosis of TAO. Interestingly, compared with the expert cohort, the DL ensemble model had higher accuracy (86% vs. 78%) and recall (89% vs. 58%), whereas the specificity was lower (84% vs. 90%).

In clinical practice, doctors usually spend a lot of time confirming TAO diagnosis at their first ophthalmologic visits. Even with an expert with abundant experience in orbital diseases, a TAO diagnosis can only be confirmed by the comprehensive assessment of the chief complaints of patients, ocular signs, medical history of dysthyroid, and imageological examination (Dolman, 2012). To a certain extent, the aforementioned studies indicated that the DL classifier using external ocular photographs might substitute the specialists to provide the initial diagnosis for patients with TAO and even accurately grade the activity and severity.

## Application of AI algorithms in the orbital imaging of TAO

Orbital imaging has provided substantial support since the 1980s in the clinical evaluation of TAO (Hosten et al., 1989). Computed tomography (CT) scanning and magnetic resonance imaging (MRI) hold the same importance with their own merits. CT can clearly present the degree of extraocular muscle enlargement and the condition of the optic nerve in the orbital apex. The delineated anatomy of the orbital wall and periorbital structures such as adjacent sinuses are essential for decompression surgery design (Cubuk et al., 2018). The benefits of MRI rely on its capacity for better resolution between muscles and orbital fat, which can help identify the specific pattern of TAO without radiation (Higashiyama et al., 2017). These characteristics have promoted the widespread use of CT and MRI in TAO, and abundant image data have become the hotbed of AI algorithms.

The research team of Shanghai Jiao Tong University explored the diagnostic value of two AI models for TAO using CT and MRI images. Lin et al. (2021) constructed DL algorithms into networks A and B, which inherited from the Visual Geometry Group (VGG) network and the Residual Neural Network (ResNet). By recruiting 160 MRI images, the accuracy, specificity, and sensitivity of network A were 0.863 ± 0.055, 0.896 ± 0.042, and 0.750 ± 0.136, respectively, for differentiating between active and inactive statuses of patients with TAO. After optimizing, the sensitivity of network B improved (0.821 ± 0.021), and the AUC of both networks was 0.922. In another study, 1,435 CT scans were used for a TAO screening 3D-ResNet model training, validation, and testing (Song et al., 2021). The results demonstrated that the AUC, accuracy, sensitivity, and specificity of this AI model were 0.919, 0.868, 0.878, and 0.865, respectively. Besides, the performance of this screening algorithm was also satisfactory in the diagnostic test.

Hanai et al. (2022) focused on extraocular muscle (EOM) enlargement in patients with TAO. The proposed diagnostic system was constructed based on deep neural networks including ResNet-50 and VGG-16. A total of 371 participants were recruited in this study with their coronal scans, including about 60% for training, 20% for validation, and the remaining 20% for test data. The results showed that the AUC, sensitivity, and specificity of this model for detecting EOM enlargement were 0.946, 92.5%, and 88.6%, respectively, indicating that the thickness of EOM could be detected using AI algorithms with high accuracy and speed in TAO.

Lee et al. (2022) developed a convolutional neural network–based model to assess the severity of TAO by analyzing the axial, coronal, and sagittal planes of CT images. A total of 288 CT images comprised mild TAO, moderate-to-severe TAO, and normal controls, which were divided into four comparable groups. Compared with controls, the diagnostic AUC of this model was 0.979 ± 0.020 for moderate-to-severe TAO, 0.895 ± 0.052 for mild TAO, and 0.905 ± 0.029 for three comparisons. The performance of the proposed model was also better than that of VGG-16, GoogleNet, and ResNet-50, and even of three oculoplastic specialists.

DON is significant with respect to the vision-threatening condition in TAO (Saeed et al., 2018). The optic nerve is suppressed by pathologically thickened tissues in the orbital apex, leading to several symptoms such as blurred vision, decreased color vision, and defect of field vision (Victores and Takashima, 2016). Early detection and intervention improve the prognosis. A hybrid model based on a deep convolutional neural network was proposed to predict DON using CT scans (Wu et al., 2022). In this model, a specific module was used to preprocess the image and extract the meaningful features for DON pathologies. The samples were divided into 87 healthy controls and 91 patients with TAO, including 42 patients with DON. After training and testing, the accuracy, specificity, sensitivity, and F1-scrore were 96%, 99.5%, 94%, and 96.4%, respectively. In this study, a DL model displayed significant advantages in predicting DON in patients with TAO.

The orbital CT scans and MRI images are the most common images examined in patients with TAO, as they can be not only evaluated by radiologists and ophthalmologists but also preprocessed into available data and then submitted to AI algorithms for further screening or predicting. The diagnosis, activity and severity grading, and DON prediction all have important clinical implications for patients with TAO patients, and AI algorithms, especially DL models, can provide satisfactory assistance to optimize this complex process in the future. The summarization of aforementioned studies in diagnosis and grading of TAO is presented in Table 1.

**TABLE 1:** AI algorithms in diagnosis and grading of TAO.

Authors (Year)	Task	Input data type	Samples dataset	AI model	Accuracy	AUC
Grus et al. (1998)	Diagnostic classification of TAO	IgG autoantibody repertoires	Sera TAO: 16, controls: 11	The probalistic neural network	96.3%	-
Salvi et al. (2002a)	Classification and progression prediction of TAO	13 clinical eye signs	246 patients with absent or inactive TAO and 152 patients with active TAO	A back-propagation neural model	Classification: 86.2%, progression prediction: 67%	-
Salvi et al. (2002b)	Classification and progression prediction of TAO	13 clinical eye signs and age, gender, smoking and follow-up interval	129 patients with absent or inactive TAO and 113 patients with active TAO, 103 normal subjects	A back-propagation neural model	Classification: 78.3%, progression prediction: 69.2%	-
Huang et al. (2022)	Diagnostic system of TAO and its special signs	Facial images	21,840 facial images from 1560 patients (3120 eyes)	ResNet-50, ResNet-101 and InceptionV3	Eye location: 0.98, cornea: 0.93, sclera: 0.87	ResNet-50: 0.91, ResNet-101: 0.92, InceptionV3: 0.89
Karlin et al. (2022)	Detecting TAO	Single front facing photograph	1944 photographs for training and 344 images for testing	ResNet-18	89.2%, 86% (compared to expert cohort)	-
Lin et al. (2021)	Detecting the active and inactive phase of TAO	Orbital MRI images	160 images from 108 patients	Deep convolutional neural network (DCNN)	0.863	0.922
Song et al. (2021)	Screening TAO	Orbital CT images	1,435 CT scans from 193 patients and 715 healthy subjects	3D-ResNet	0.868	0.919
Hanai et al. (2022)	Detection of EOM enlargement in TAO	Orbital CT images	371 participants (60% for training, 20% for validation and 20% for testing)	ResNet-50 and VGG-16	-	0.946
Lee et al. (2022)	Diagnosis and severity evaluation of TAO	Orbital CT images	288 CT scans from 200 patients and 100 controls	CNN	Mild TAO: 0.826, moderate-to-severe TAO: 0.930, three comparisons: 0.842	0.8950.9790.905
Wu et al. (2022)	Prediction of dysthyroid optic neuropathy (DON) in TAO	Orbital CT images	178 participants (42 DON, 49 TAO without DON, 87 controls)	DCNN	96%	-

EOM, extraocular muscle; DCNN, deep convolutional neural network; DON, dysthyroid optic neuropathy.

## Application of AI algorithms in treating TAO

### GC pulse therapy

In accordance with the 2021 EUGOGO guidelines (Bartalena et al., 2021), intravenous GCs combined with mycophenolate sodium were nominated as the first-line treatment for moderate-to-severe and active TAO. The pulse therapy of GCs has been used in TAO management for decades, and many studies have demonstrated substantial benefits. Still, about 20%–30% of patients in clinical trials were unresponsive to GC treatment, even with unbearable adverse effects (Vannucchi et al., 2014; Zhu et al., 2014). The general method in a clinic is closely monitoring the initial outcomes of GC treatment, which determine the subsequent remedies, to avoid the unworthy risk of overdosed GCs. Thus, a practical method for response prediction before GC therapy is required.

Coronal T_2_-weighted MRI images with fat suppression can clearly show the cross-sectional morphology and radiomics features of EOMs. Hu et al. (2022) developed three ML-based models to analyze the radiomics data of patients with TAO. In this retrospective study, 110 samples were selected, and GC-responsive (*n* = 62) and unresponsive (*n* = 48) cases were equally split into training and validation sets. A semi-quantitative imaging model was also built by two experienced doctors, in which the absolute signal intensities of EOMs were manually measured and normalized to values of ipsilateral temporal muscle. The AUCs of the three ML-based models in two sets (0.968 and 0.916; 0.933 and 0.857; 0.919 and 0.855) were all better than the performance of the semi-quantitative method (0.805). Additionally, including the disease duration of TAO into AI algorithms enhanced the diagnostic ability in their validation (AUC: 0.952 vs. 0.916), indicating the advantage of the AI model in predicting the response of patients with TAO to GCs.

Besides the use of MRI, a prospective and observational protocol was proposed by Wang et al. (2021) for developing a new prediction model. A total of 278 untreated patients with moderate-to-severe and active TAO will be recruited into this trial based on the events per variable method and previous models. The clinical data and AI-related parameters will be collected from these volunteers before their standard 12-week GC pulse therapy. After treatment, the patients will be divided into GC-responsive/unresponsive groups based on their outcomes of therapy. The facial morphological changes and traditional clinical data will be used to develop a new AI model, which can recognize the best variables for GC-response prediction. This study is an ongoing project, and the findings can guide on the individualized GC treatment for TAO.

### Orbital radiotherapy

Orbital radiotherapy in alliance with GCs was recommended as the second-line treatment (Bartalena et al., 2021). The therapeutic effect of regional irradiation, which seems to have a mutual promoting effect with GCs (Bartalena et al., 1983; Oeverhaus et al., 2017), was demonstrated by several randomized controlled trials in TAO (Prummel et al., 2004). Conventionally, a low dose of 20 Gy was given for about 2 weeks (Tanda and Bartalena, 2012). Although adverse events were relatively rare in orbital radiotherapy (Marcocci et al., 2003), the irradiation target still needs to be precisely delineated to avoid possible damage to organs at risk (OARs).

Jiang et al. (2021) developed a DL model based on a fully convolutional network (FCN) to realize the auto-segmentation of the clinical target volume (CTV) for patients with TAO. Briefly, CT images from 121 patients with TAO undergoing radiotherapy were collected for training and testing. The outcomes were set as the Dice similarity coefficient (DSC) and Hausdorff distance (HD). Because of two orbits, Jiang et al. suggested treating the two-part CTV as one target, which was demonstrated to have higher HD values than the separate method (8.23 ± 2.80 vs. 9.03 ± 2.78). The dosimetric comparison showed that both algorithms based on the FCN model performed better than manual segmentation. In another study (Jiang et al., 2020), a stacked neural network using adjacent anatomy for target location was proposed to improve the accuracy of CTV. Compared with the FCN model, this stacked network increased the bilateral DSC by 1.7% and 3.4%, but reduced the HD value by 0.6.

Position errors caused by manual or mechanical misconduct are probable in the actual delivery, except for planned contours before irradiation (Ezzell et al., 2003). The electronic portal imaging device (EPID) dosimetry was established for real-time supervision. Zhang et al. (2021) conducted an interesting study for integrating EPID measurements and AI algorithms. First, the irradiation plans were duplicated from 40 patients with TAO to a solid head phantom, and position errors combined with varying translation errors in different directions were added to the protocols. The radiomics of EPID measurements were extracted and analyzed using 3 ML models. Their AUC values were all above 0.90 for position error detection and relatively lower (0.76, 0.80, and 0.91) for direction identification. The research team classified all the position and direction errors into three types (Dai et al., 2021). The aforementioned ML models plus a CNN model were also applied to recognize these errors using radiomics data from EPID transmission maps as inputs. The classification accuracies of the CNN model performed well in this competition. Additionally, Liu et al. (2022) developed a deep neural network (DNN) algorithm with structural similarity difference and orientation-based loss, which could provide more features and information from EPID images. A total of 2240 EPID fluence maps were enrolled and subjected to the DNN model for training and testing. The proposed model outperformed with a better prediction accuracy (0.722) than other ML models and previous study results.

The OARs contain lenses, optic nerves, retina, and lacrimal glands during orbital radiotherapy. AI-based algorithms can optimize the procedure of restricted irradiation and reduce the potential risks, which may be beneficial for TAO treatment. Other orbital diseases requiring radiotherapy, such as mucosa-associated lymphoid tissue lymphoma and optic nerve sheath meningioma, may also benefit from AI applications.

### Orbital decompression surgery

Orbital decompression surgery was introduced to solve the conflict between excessive orbital contents and relatively inadequate orbital volumes by removing parts of the orbital bony wall and fat (Roncevic and Jackson, 1989). This surgery would serve as a salvage operation only for uncontrollable exposure keratopathy or DON with unresponsive GCs (Bartalena et al., 2021). It performs during a later course of TAO management, when patients step into the inactive phase with stable disfigurements (Limone et al., 2021).

Yoo et al. (2020) introduced a generative adversarial network (GAN) model to predict postoperative appearance before decompression surgery. A GAN could automatically synthesize medical images by a generator module, which learns to map samples from a random distribution to the specific distribution (Iqbal and Ali, 2018). This transformation was conducted based on the preoperative facial images. In brief, 109 pairs of matched images were augmented for the proposed GAN model training. These AI-synthesized images were semblable after their evaluation compared with the actual postoperative facial images, whereas the image quality was unsatisfactory. Besides, an additional training set, containing 76 paired datasets and 1000 GAN-generated datasets, was used to enhance the ability of the DL classifier (based on VGG-16) for TAO identification (AUC, 0.872 vs. 0.957). The overview of discussed studies in treatment of TAO is exhibited in Table 2.

**TABLE 2:** AI algorithms in treatment of TAO.

Authors (Year)	Task	Input data type	Samples dataset	AI model	Accuracy	AUC
Hu et al. (2022)	Prediction of therapeutic response to GCs in TAO	Orbital T_2_-weighted MRI images	Training (n = 78) and validation (n = 32) cohorts	LRDTSVM	-	0.968, 0.9160.933, 0.8570.919, 0.855
Wang et al. (2021)	Developing a prediction model for identifying intravenous GCs response	Traditional clinical information and PPVs output by four AI models	278 untreated patients with moderate-to-severe and active TAO	Ongoing study	Ongoing study	Ongoing study
Jiang et al. (2021)	Auto-segmentation of CTV for TAO patients	Orbital CT images	121 patients undergoing radiotherapy	FCN	-	-
Jiang et al. (2020)	Improving the auto-segmentation accuracy of CTV in TAO	Orbital CT images	120 cases with moderate-to-severe TAO	Stacked neural network	-	-
Zhang et al. (2021)	Detecting positioning error in TAO radiotherapy	Radiomics analysis from EPID	Treatment plans of 40 patients with TAO	SVMKNNXGBoost	-	Positioning errors: all above 0.90; direction classification: 0.76, 0.91, 0.80
Dai et al. (2021)	Identifying positioning error in TAO radiotherapy	Radiomics data from EPID transmission maps	40 TAO patient radiotherapy plans	SVMKNNXGBoostCNN	ML 1: 0.532-0.889ML 2: 0.491-0.949ML 3: 0.671-0.931CNN: 0.689-0.949	ML 1: 0.778-0.945ML 2: 0.682-0.989ML 3: 0.779-0.990CNN: 0.832-0.992
Liu et al. (2022)	Position error classification in radiotherapy of TAO	EPID fluence maps	2240 EPID fluence maps	DNN	0.722	-

LR, logistic regression; DT, decision tree; SVM, support vector machine; PPV, positive predictive values; FCN, fully convolutional network; EPID, electronic portal imaging device; KNN, k-nearest neighbors.

## Application of AI algorithms in privacy safeguard of TAO

The physiognomic changes in patients can be crucial for a real-time evaluation of the disease stage in the clinical diagnosis and management of TAO. The storage of facial images is important, which can also be used in AI training as mentioned earlier (Huang et al., 2022; Karlin et al., 2022). The facial privacy of patients was commonly anonymized by cropping images into a restricted area in the overwhelming majority of data collection and literature reports. Regarding ophthalmology, the retained field generally ranged from the supraorbital arch to the infraorbital margin. However, this pattern could not elude advanced facial recognition, while dropping some meaningful clinical information (Clover et al., 2010).

Recently, a creative study on AI-assisted privacy protection was published in *Nature Medicine*. Yang et al. (2022) introduced a novel technology named the digital mask. This mask could be synthesized with diagnostic information and without recognizable characteristics in the original face depending on DL algorithms and three-dimensional reconstruction. They carried out a prospective clinical trial to evaluate the feasibility of this mask. A total of 420 patients (from departments dealing with strabismus, pediatric ophthalmology, TAO, and oculoplasty) were recruited, and 253 were confirmed with associated ocular diseases through facial diagnosis. According to their results, all the pixel errors in eyeball and eyelid reconstruction were about 1%. Cohen’s κ values between 12 ophthalmologists and digital masks demonstrated high consistency (*κ* = 0.801 for TAO and 0.845–0934 for other diseases). In the recognition-removal experiments, the accuracy of recognition by respondents between cropped pictures and masked images was 91.3% *versus* 27.3%. Regarding AI recognition systems, Rank-1 was <0.02 for the three AI models, indicating the extremely low possibility for the correct identification of digital masked images. Besides, Yang et al. also investigated the willingness of patients to share facial images, and the result confirmed that the proposed digital mask did help.

## Discussion

AI applications occupy an increasing important part in clinical practice owing to their rapidity, precision, and economy. In ophthalmology, many AI applications have achieved satisfactory performance in diagnosing and predicting several retinal diseases based on the contribution of widely used fundus images (Li et al., 2018b; Nagasato et al., 2018; Peng et al., 2019). Unlike the majority of ocular diseases, TAO is more specialized and has gained the attention of fewer ophthalmologists, implying inadequate medical resources for such patients. The burgeoning AI represents a promising future for solving this problem.

The diagnosis and grading of TAO are highly comprehensive, including the summarization of chief complaints and symptoms, examination of external ocular signs, detection of thyroid function and immunology, and assessment of orbital images (Smith and Hegedüs, 2016). Facial images can be easily acquired using smartphones, and automated AI algorithms can help identify meaningful signs and provide diagnostic advice. Orbital CT and MRI scans are broadly used, and the conventional images can be converted into precise data for AI analysis, thus avoiding variable subjective interpretation between observers. The response to GC therapy and the occurrence of DON can also be predicted by AI-aided image processing with digital standards.

Among these aforementioned studies, we found that developing AI models to predict the postoperative appearance of orbital decompression may worth more discussion. In a recent study, Wickwar et al. (2018) conducted a qualitative study about patients’ expectations of orbital decompression surgery. It found that the inability to completely imagine post-operative appearance caused some anxieties, which may be greatly ameliorated by AI-synthesized images. On the other hand, there were different feelings on whether outcomes of surgery had met patients’ expectations. And possible strabismus or asymmetry may worsen the situation (Del Monte, 2002). Thus, it would be more reasonable for this kind of AI-assisted prediction to take these factors into consideration. Overall, predicting postoperative appearance by AI models does help propagandize orbital decompression but still needs to be improved.

AI development in TAO has some challenges. Firstly, the incidence of TAO can hardly be comparable to other ocular diseases, especially cataracts and diabetes retinopathy (Shah and Patel, 2022). CT and MRI examinations are also not as simple as fundus photography and optical coherence tomography. Attributed to these two factors the sample size of TAO-related data is relatively low, substantially hindering the advance of AI models in this field. Secondly, the exophthalmometry values and orbital depths are significantly different between races (de Juan et al., 1980; Tsai et al., 2006), implying that the AI model trained based on Caucasian data may not be practicable for Chinese Asians, and extra data collection is needed. Thirdly, some common problems still exist. Many clinicians are reluctant to use AI models in their practice due to the lack of understanding and trust (Maddox et al., 2019), while most patients also prefer to meet a doctor in reality (Keel et al., 2018). The AI-relevant laws and social supervision cannot match the present technology. Under this situation, we suggested that a TAO-related database collaborated by domestic and international centers would play a vital role in AI development. Establishing some AI pilot schemes in expert clinics of TAO could also help the verification and generalization of AI applications in TAO. Regarding the privacy of patients, the novel introduced digital mask (Yang et al., 2022) can provide us an admirable start to build the safeguard.

**FIGURE 1:**
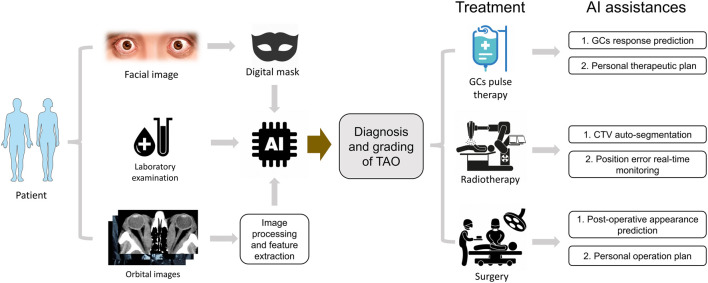
The hypothetical pattern of AI applications in TAO clinical practice. The diagnosis and grading of TAO could be deduced by an integrated AI module based on masked appearance, laboratory index and processed orbital images. The different therapy options could be optimized by AI assistances automatically.

Although several challenges and problems stand in the way of AI development in TAO, we still need to embrace this promising technology. For further studies, it is foreseeable that the integration of AI models using clinical signs and orbital images can create more reliable AI-based systems for TAO diagnosis. Using AI algorithms, we may separate the standard 12-week GC therapy and record changes from intervals. The AI prediction for GC response can be more precise with these data and help formulate the individual treatment options for each patient with TAO. Through all-around integration, the future scenario of AI applications in TAO may develop as the flow chart in Figure 1.

## Conclusion

In summary, the emerging AI algorithms may potentially improve the accuracy of TAO diagnosis and reduce the economic costs for patients to access qualified healthcare resources. This automated technology can instantly help optimize therapeutic strategies and surgical design during the long course of TAO management. We believe that AI algorithms may become vital in TAO clinical practice soon with the continuous accumulation of TAO data and an improvement in computing capacity.
